# The face as folded object: Race and the problems with ‘progress’ in forensic DNA phenotyping

**DOI:** 10.1177/03063127211035562

**Published:** 2021-08-02

**Authors:** Roos Hopman

**Affiliations:** University of Amsterdam, Amsterdam, The Netherlands

**Keywords:** forensic DNA phenotyping, temporality, race, data, praxiography, temporal folds

## Abstract

Forensic DNA phenotyping (FDP) encompasses a set of technologies aimed at predicting phenotypic characteristics from genotypes. Advocates of FDP present it as the future of forensics, with an ultimate goal of producing complete, individualised facial composites based on DNA. With a focus on individuals and promised advances in technology comes the assumption that modern methods are steadily moving away from racial science. Yet in the quantification of physical differences, FDP builds upon some nineteenth- and twentieth-century scientific practices that measured and categorised human variation in terms of race. In this article I complicate the linear temporal approach to scientific progress by building on the notion of the folded object. Drawing on ethnographic fieldwork conducted in various genetic laboratories, I show how nineteenth- and early twentieth-century anthropological measuring and data-collection practices and statistical averaging techniques are folded into the ordering of measurements of skin color data taken with a spectrophotometer, the analysis of facial shape based on computational landmarks and the collection of iris photographs. Attending to the historicity of FDP facial renderings, I bring into focus how race comes about as a consequence of temporal folds.

## Introduction

[A]nthropometry is virtually gone. Typology is gone. Craniology, with its indexes and skull types, is gone too. And gone for good is old-fashioned anomaly-anatomy, once a respectable mainstay of anatomical physical anthropology. Today’s physical anthropology leans heavily upon today’s technology. Measuring is done – but on radiographs, using automatic print-out, motor-driven equipment. Reflection colorimeters and spectrophotometers have replaced colored tiles and dyed-hair samples … the genetical theory of natural selection, biochemical genetics, biophysical research methods, and the experimental approach freed American physical anthropology from its last-century technical and intellectual fetters. (Garn, 1962: 917–918)

In this excerpt from a 1962 essay by the physical anthropologist Stanley Marion Garn, two modes of progress coincide: *scientific* and *societal*. On the one hand, Garn heralds the then-novel technological advancements in physical anthropology. Referring back to the title of a pivotal 1951 article by Sherwood Washburn, the ‘new’ physical anthropology would be a ‘complete departure from the historical past’ (Garn, 1962: 918). ‘Old’ methods such as anthropometry, topology and craniology would be left behind with the past ‘for good’ and replaced by innovative technologies. On the other hand, scientific progression beyond ‘pre-genetical typological approaches’ coincides with the releasing of the ‘intellectual fetters’ of race (Garn, 1962: 918; Radin, 2013: 487). The new physical anthropology would do without race, which in Garn’s words had been holding back the progression of the field into a ‘newly exciting’ age (Garn, 1962: 918). In his 1951 paper, Washburn had pointed to a similar shift. He emphasised that physical anthropology should move away from racial science and, taking inspiration from human genetics, should shift its focus towards populations (Washburn, 1951; see Haraway, 1988; Marks, 1995; Radin, 2013: 487).

The interventions of Garn and Washburn suggest that race, a problematic element in physical anthropology, could be left behind through technological and intellectual innovation. Their essays invoke a progressive and modernist trajectory of technological and societal development. On to this view, technology steadily advances in an ‘irreversible line’, ‘leaving behind … a trail of errors finally corrected’ (Serres and Latour, 1995: 48). Findings from the emerging field of genetics were crucial here, as they demonstrated that humans are not easily divided into separate races. Garn’s paper therefore reflects a broader trend in studies of human diversity that gained traction during the second half of the twentieth century, when interest shifted away from typology and increasingly towards the study of genetic populations (e.g. Cavalli-Sforza and Bodmer, 1971). This did not mean, of course, that race became irrelevant to studies of human genetic difference (see, e.g. Lipphardt, 2012; Reardon, 2005).

Fifty-six years after the publication of Garn’s paper, in late 2018, I visited a German university to attend a workshop on the statistical analysis of 3D shapes, particularly of facial form. I attended because I was conducting research on forensic DNA phenotyping (FDP), an emerging forensic genetic technology that can be used to generate clues about the appearance of an unknown suspect by predicting physical traits from DNA traces. At the workshop, organised in collaboration between the anthropology, statistics and mathematics departments, I was hoping to learn about the most recent developments in the prediction of facial morphology from DNA. I arrived just before it started and had the opportunity to informally meet some of the other participants: a small group consisting of a mathematician, a physical anthropologist, a computational scientist and a geneticist. We gathered early on a Monday morning, eating cookies and sipping coffee as we sat around a table. Our host asked us to introduce ourselves and our work. I started to give a brief synopsis of the project I was working on. Upon uttering the word ‘race’, however, the computer scientist abruptly interrupted my introduction. Surprised, he stepped in to tell me that ‘biological race does not exist’: ‘Plants and animals classify into clear-cut clusters in data. For humans this is not possible. We have a lot of admixture, there is a gradual transition from one population into the other’. He stressed that ‘modern methods are moving further and further away from race’.

In line with Garn, this scientist presented race as something of the past, as something that can be left behind through methodological advancements. The linear narrative of scientific development that underlies this comment is telling of research into human genetics more generally, where, as pointedly noted by Rothman (1998: 25), ‘time seems to come in only in discussions of evolution, of “progress”’. After his remark on race and modern methods, the computer scientist went on to speak about a facial reconstruction he had been involved in. The problem with this reconstruction, he explained, was that the skull (of which only the upper cranium was intact) had been given a lower jaw that was ‘too Western European’. He had solved the problem, he went on, by reconstructing a ‘more Mongoloid’ jaw.

This anecdote signals a paradox that upsets the progressive, linear temporality and the gradual move beyond race addressed above, and raises questions about progress, time and race in this field. In this article I seek to address this paradox and complicate a linear temporal approach to scientific progress through the case of forensic DNA phenotyping (FDP). FDP is an emerging field of forensic genetic research in which the bodily surface is gaining renewed salience. By establishing associations between genotypic and phenotypic data, attention is shifting back towards physical variation between human groups. Through this shift, physical anthropological knowledge is made relevant in novel configurations. This is not necessarily apparent from facial renderings produced through FDP, but becomes evident in the data practices that inform the research behind them. In this article I do not focus on the application of FDP, but on the development of these technologies themselves. I build on the concept of the fold (Serres and Latour, 1995), the folded *object* in particular (M’charek, 2014) to show that in these data practices ‘history is never left behind’ (p. 31). I demonstrate what is ‘folded in, folded away, or unfolded’ (Bauer, 2015: 156) in three FDP research practices revolving around the collection, ordering and analysis of data on human biological variation, pointing out how race becomes part of these practices.

### Folded faces

Race is typically quickly dismissed as irrelevant to discussions on the most recent developments in studies of facial genetics. As Skinner (2006: 461) points out, ‘the suggestion is that science’s involvement in racialization has long passed, its outdated formulations living on only in the misguided and ill-informed assumptions of the prejudiced’. Yet multiple authors have shown how race is implicated in FDP technologies. Most importantly, because phenotypic clues that are generated through FDP are generic, they do not point the investigation towards individual suspects but to suspect *populations* (Cole and Lynch, 2006; M’charek, 2008; Ossorio, 2006; Skinner, 2020; Wienroth et al., 2014). Results might, for example, report a suspect is likely to have dark eyes and hair, or that he is most probably of Southern-European descent. Because these results are generic they risk feeding into ‘folk notions’ of race (Ossorio, 2006).

It is, however, believed and promised that with the advancement of technology and the collecting of increasing amounts of data FDP will be able to gain specificity and *over time* predict individual facial composites: ‘Clearly, being able to predict individual-specific faces from DNA would be the ultimate goal of FDP and the dream of police men and women’ (Kayser, 2015: 44; see also Crouch et al., 2018: E676). FDP practices are therefore importantly *data* practices. Building on larger sets of more precise data would allow researchers to move beyond the production of racialised population averages and towards the characterisation of individual-specific phenotypes (see Hopman, 2020), leaving behind associations with race and racial science.

In this article I contribute to these discussions on race and FDP by attending to time and temporality, investigating the ‘historicity’ (M’charek and van Oorschot, 2019: 242) of FDP practices. Research practices that inform the production of ‘DNA-based’ facial renderings *fold* into themselves particular histories, technologies, objects and also futures. I argue that these facial renderings are therefore ‘multitemporal, simultaneously drawing from the obsolete, the contemporary, and the futuristic’ (Serres and Latour, 1995: 60). In this paper I bring these ‘folds’ into view, building on M’charek’s (2014) analysis of the mitochondrial DNA reference sequence. The folded object draws on a topological version of time that Serres describes as a ‘crumpled handkerchief’ (Serres and Latour, 1995: 60). The handkerchief, when crumpled, gathers together points that would otherwise remain distant. As such, the folded object allows for a conception of time that is not linear but is configured in intricate ways in objects. Folded time is topological in the sense that it can make the distant near. It does not progress from one point to another in a straight line but is unstable and unpredictable (Serres and Latour, 1995: 58). Like the crumpled handkerchief, facial renditions produced through FDP make adjacent practices from different places and times. In the application of contemporary technologies to phenotypic data, nineteenth-century statistical averaging techniques as well as physical anthropological measuring and data-collection practices resurface. In this article I articulate these histories and point out when race becomes of relevance.

In considering ‘DNA-based’ facial renderings as folded objects I also draw on the work of Bauer (2015), who takes up M’charek’s concept to think through the complexities of Soviet human genetics. She focuses on the genogeographical atlas as an object that draws together a century of scientific research, techniques and particular political epochs and folds these in the visualisations of maps (Bauer, 2015: 148). Because the maps enable exploration of these different times and places, Bauer (2015) argues that ‘genogeographic maps function as time machines … we can account for the entangled temporalities and the things folded in, folded away, or unfolded, as part of map-making’ (p. 154, 155–156). Likewise, I aim to show the particular pasts and presents folded together as part of *face-making*.

The topological approach to time that I take in this paper is especially helpful when thinking through an object like race, which comes ‘in many different guises’ (M’charek et al., 2014: 462). This is not to accuse my subjects of racism. Rather, I zoom in on their data technologies and practices, and unravel how they depend on technologies and methods from earlier times and practices.

The fold, finally, allows for a consideration of anticipated futures: FDP is importantly an anticipatory technology (Fortun, 2005; Selin, 2008; Wienroth, 2018) that ultimately promises individuality. As I show below, the realisation of this future requires relying on particular pasts. How these pasts are implicated in this future imaginary, and in the laboratory presents I encountered, will become clear through my analysis of three cases.

### Ethnographic fieldwork and a praxiographic approach

I combine a theoretical sensitivity to time and the fold through a praxiographic approach, which implies paying attention to practices and ‘the way objects are enacted in practices’ (Mol, 2002: vii). In particular, I attend to how the object of race is enacted in ‘DNA-based’ face-making practices. I build on ethnographies conducted over the course of half a year, during the winter of 2017–18, in various laboratories: two in Europe and one in the United States. The first of the European labs was geared towards research, specialising particularly in the development of novel FDP tools for the prediction of pigmentary traits. The second European lab focused less on research and more on the routine application of forensic analyses, including kits for the prediction of biogeographical ancestry, an analysis also described as ‘indirect phenotyping’ (Koops and Schellekens, 2008: 211). The North-American lab was again a research lab, conducting research on associations between genotype and phenotype. I furthermore draw on fieldwork conducted at international academic conferences and workshops on forensic genetics, and publications on FDP in forensic genetic journals.

The cases that I introduce in this article stem in particular from research at the North-American lab, which I visited in early 2018 accompanied by my colleague, Amade M’charek. We spent two intensive days at the laboratory, during which we were shown around by the head of the lab and his PhD students; we also had informal conversations with the lab staff. We were introduced to lab equipment, data storage devices and technologies used to collect data on human variation. We encountered high resolution cameras, a dynamometer, spectrophotometers, scanners, 3D software and devices to measure hand- and foot morphology.

The data practices we encountered during our tour in this research lab seemed far removed from the facial renderings that FDP generates, yet they are fundamental to producing them. Ultimately, the head of the lab told us, he was aiming to map ‘the whole face genetically’, enabling the prediction of complete faces from DNA. In order to achieve this goal, the researchers at this lab sought to establish associations between the phenotype and the genotype. They performed large-scale statistical analyses comparing data on phenotypic traits to genetic markers, aiming to identify genetic markers that affected facial appearance. For these analyses to be informative, however, large quantities of data covering a wide variety of phenotypes were necessary. In this lab researchers were thus working on the *collection, ordering* and *analysis* of data on phenotypic variation.

In the following I will walk through the lab as we were walked through it by the head of the lab and his PhD students, pausing with three facial traits along the way. First, I bring into focus the skin. Researchers measure skin pigmentation using a spectrophotometer; the ordering of these data reproduces familiar methodological problems and classifications. Second, I move on to facial shape. I bring into view a process of facial landmarking that maps the facial surface and its morphologies. Here I show the increasing precision, detail and speed with which faces can be processed, yet in the analysis of these facial data the effects of particular past averaging practices resurface. In the final case I pause with the collection of iris photographs to redirect our attention from a focus on the past to a focus on the future of these technologies, providing the broader contours for the other two cases.

## Ordering: Skin pigmentation

**Figure 1.**
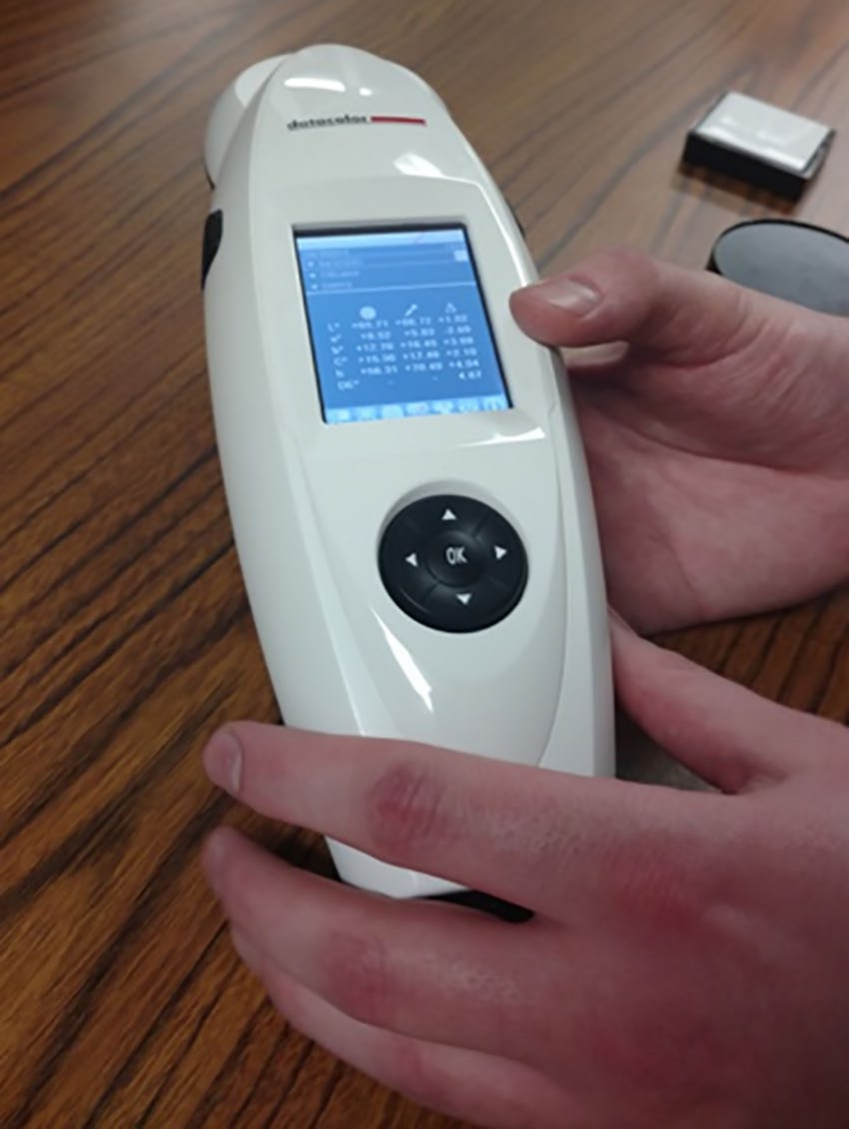
Spectrophotometer and calibration tiles.

The PhD students take us to look at instruments they use to measure and record different kinds of physical variation. They for example take out a spectrophotometer, a device used to measure skin pigmentation (Figure 1). When measuring the pigmentation of the skin they place the instrument on three different locations on the body: the underarms (because this skin has limited exposure to the sun), the forehead (frequently exposed to the sun), and the top of the hands. By including these different patches of skin, they can get an idea of a subject’s tanning ability. The PhDs encourage me to try it out, and so I use the device on myself, placing it on the skin of my underarm. One of them gives me instructions: ‘You have to make sure your skin is really trapped beneath the machine, there can’t be any other light on your skin. And you have to avoid moles and veins, or else you get false readings.’ I press the button I am instructed to push down. The spectrophotometer subsequently ‘flashes a bright light’ and then ‘measures the amount of light that bounces back’, as one of the PhD students narrates. After the light has flashed, the device’s display presents me with a list of numbers. They appear to be of high specificity and I cannot make sense of them. I ask what the numbers mean, and am explained that they are based on calibration with a white and a black ceramic tile. These are its references. Based on the color of these two tiles, the measured skin is attributed a value. It is put somewhere on a scale between the two extremes, ordering it numerically. Findings produced with the spectrophotometer are complemented by skin color data based on ‘self-reported identity’ information sheets that participants fill out. Yet the PhD students tell me they consider the spectrophotometer the ‘best’ and ‘most objective’ way of measuring skin pigmentation.

The first physical characteristic I want to take into consideration is the skin. Of the pigmentary traits targeted for prediction from DNA, besides hair- and eye color, genetic knowledge of skin color is the least developed (Walsh et al., 2017: 847). Only recently did geneticists start to uncover the trait’s great genetic variation: ‘the historical bias in favour of genetic studies in European and European-derived populations has blinded us to the magnitude of pigmentation’s complexity’ (Quillen et al., 2019: 4). In the Netherlands, a tool to categorically predict skin color has now been validated for implementation in criminal cases. The first of its kind to be forensically validated, this tool predicts skin color in five categories, ranging from ‘Very Pale’ on one end to ‘Dark-to-Black’ on the other. In the following I focus specifically on the production of these categories, a process of translation in which the reflectance values measured with the spectrophotometer are *ordered*, and the historical classifications this translation reproduces.

As described in the vignette above, the spectrophotometer measures the amount of light that is reflected by the surface of the skin and translates this into ‘reflectance values’ through calibration with two ceramic tiles. Pigmentation is quantified, allowing for the variations in pigmentation to be organised numerically on a continuous scale from the lowest to the highest value. This process resembles Latour’s (1999) description of scientific referencing in soil studies, or pedology. Like the use of the Munsell code in this field, the ceramic tiles provide a reference for arranging ‘all the nuances of all the colors of the spectrum by assigning each a number’ (p. 58). Yet because the reflectance values are highly specific and particular to each individual that is measured, some form of categorisation is needed to make the numerical data ‘manageable’ (Daston and Galison, 1992: 85). A particular range of values is therefore classified into category A, another in category B and so on. Such an ordering process is described in the methods section of a study on skin pigmentation conducted by Maroñas et al. (2014: 36):

In order to obtain a link between the continuous range of readings from the spectrometer to categorical classifications of skin colour, *more akin to eyewitness*, we matched the instrument value ranges to a simplified set of categories. Four volunteers (not dermatologists) classified the skin colour of photographs, without guidance or cues, into white, intermediate or black. [emphasis added]

The reflectance values were classified through the visual assessment of volunteers, drawing the categories of ‘white’, ‘intermediate’ and ‘black’ into the research. The introduction of these particular categories is significant, as they ‘valorize some point of view and silence another’ (Bowker and Star, 1999: 5). As argued by Bowker and Star (1999), the choice among sets of categories is always an ethical one, and deciding on which categories to use carries ‘serious material force’ (p. 6) In this particular study, the decision to articulate differences in skin variation through the categories of white, intermediate and black is a decision that valorises everyday racial categories over more nuanced possibilities. Furthermore, through building upon the visual judgement of ‘non-experts’, particular histories of ordering skin color are brought into proximity.

To demonstrate this historicity I would like to pause with visual assessment, as it has a long history in anthropological studies of skin color. Before the introduction of spectrometry in the 1950s, visual matching was the primary means of classifying skin color in physical anthropology. Paul Broca’s ‘Table chromatique’, which worked by visually comparing the subject’s skin to a set of standardised references, was one system in wide use (Blackwood, 1930: 137). Another of these visual matching-based methods was developed in the late 1800s by Austrian anthropologist Felix von Luschan, consisting of a set of 36 glass tiles. These tiles, known as the *Hautfarbentafel*, were ordered from 1 to 36 according to their color and hue. Researchers would visually compare the subject’s skin to the standardised colours of the tiles and assort it the number that they considered most in line with their complexion. The results of these classifications subsequently fed into the distinguishing of a limited number of skin color-based human ‘types’, and their geographical distribution. In his work *Le razze e i popoli della terra*, Italian geographer Biasutti (1941), for example, used data derived from color-matching methods to demonstrate the global distribution of skin color types, dividing the world up into the categories ‘very pale’, ‘pale’, ‘medium’, ‘dark’ and ‘very dark’. Notably, these categorisations are directly reflected in the forensic tool applied in contemporary Dutch investigations.

The results of the tile method were, however, hard to reproduce (Jablonski, 2004: 591). The material of the tiles produced glare and typically contained physical imperfections. Furthermore, complained British anthropologist Blackwood (1930: 138), ‘the polished surface … does not bear the remotest resemblance to the surface of the human skin’. In addition, the assessment depended on the particular researcher doing the matching, which introduced further problems concerning the standardisation of the data (Swiatoniowski et al., 2013: 325). These issues were considered problematic because they took away from the ultimate purpose of the method: to move away from verbal description of skin color and towards standardised data. Furthermore, the 36 tiles were considered ‘quite inadequate to cover the enormous range required to measure the varieties of human skin’ (Blackwood, 1930: 138). With the introduction of reflectance spectrometry in the 1950s these color matching methods were therefore quickly abandoned (Jablonski, 2004: 591). Interestingly, with von Luschan’s glass tile method, anthropologists sought to escape subjectivity by moving away from verbal description and standardising skin color measurements, only to run up against the subjectivity of the human observer. Likewise, researchers producing numerical results through reflectometry still needed to revert to ‘untrained’ vision to attribute reflectance values an everyday meaning.

Material traces of the tile-based comparison become evident in the calibration of the spectrophotometer. In the lab where we received our tour, two tiles functioned as the references against which the spectrophotometer was calibrated. Although differently configured, the tiles remain the reference in both cases, and additionally feed into the production of similar results.

Historical data produced through von Luschan’s method are deemed invaluable in present practices, so much so that researchers are seeking ways to make them compatible with contemporary skin pigmentation data. In this lab, a formula was generated that could convert data derived by the tile method, deemed ‘irreplaceable’, into a format compatible with modern data. To do so, two human observers were assigned the task of matching the skin of 246 study participants to the tiles numbered from 1 to 36, putting von Luschan’s method back into practice. Through this conversion, direct material traces of the von Luschan tiles made their way into the lab. The skin that was taken for comparison was to ‘reflect both historical and contemporary skin color study protocols’ (Swiatoniowski et al., 2013: 326). Here, the past was deliberately mobilised towards contemporary analysis, folding together ‘historical’ and ‘contemporary’ practices through the data conversion. Continuities can therefore be located in the technologies used to generate and order data, but also in the data themselves.

Today, geneticists do not aim to find physical proof of biological races, nor to use skin pigmentation data to differentiate human types. Yet the above demonstrates that race re-appears in the categorisation work aimed at ordering measurements in studies on the genetics of human skin pigmentation. As Latour (1999: 38) reminds us: ‘the word “reference” comes from the Latin *referre*, “to bring back”’. The ceramic tiles-based referencing method and subsequent translation of reflectance values into skin color categories, then, can be argued to *bring back* the visual assessment crucial to historical studies of the skin. In doing so they bring with them their methodological problems, generating classifications that directly reflect, for example, Biasutti’s racial divisions of skin color distribution. Von Luschan’s method and Biasutti’s skin color distribution maps are seen as irreplaceable ‘records of variation in skin color that existed before wide-spread globalization and cannot be measured again’ (Swiatoniowski et al., 2013: 329). As such, this skin-measuring practice works in ‘an archival mode’, ‘preserving and repurposing data from the past’ (Bauer, 2015: 152). Rather than being the latest stage in a linear development of skin color measurement, spectrometry folds these histories in itself through processes of *translation* of data into categories, *calibration* of the measuring device with tile references and the *repurposing* of historical data, reintroducing racial classifications into genetic diversity research.

## Analysis: Facial morphology

We move on to another space: a small room without daylight. It is separated into two compartments by a heavy black curtain that is attached to the ceiling. Close to the entrance of the space, a computer stands. One of the PhD students explains this is where they store the data produced in the room. We then go behind the curtain, entering the camera space. This is the ‘static setup’, the PhD student explains, as stills of faces are collected here. Participants sit on a chair in the middle of the space and face a screen in front of them. The screen is surrounded by four big lights. Furthermore, there is a cluster of three cameras attached above the screen, and another cluster of three cameras underneath it. There are more cameras on the right and left side of the lights, twelve in total (divided into four clusters of three).

**Figure 2.**
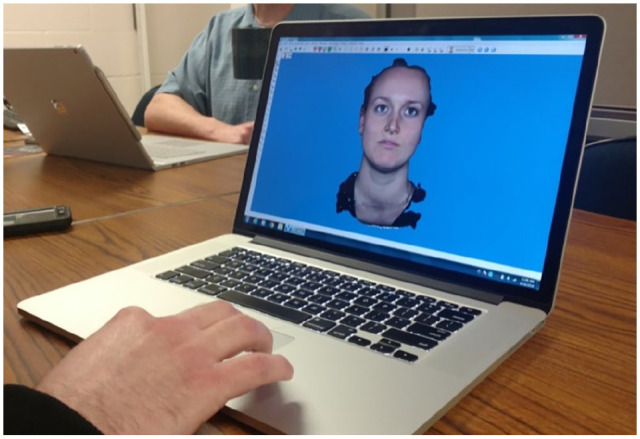
The author’s 3D digitised face.

In order to create 3D faces, photos taken with the different cameras are combined into one depiction. I am curious about this process and ask one of the PhD students if he wants to demonstrate how this works by taking photos of my face. He takes me to a part of the lab where there is less natural light and asks me to stand in front of a wall. He then tells me to keep my face in a neutral expression and to stand still as I look at a particular point behind him. He takes three pictures. We walk back over to the communal area of the lab and sit down at the table. He takes out his laptop and uploads the three pictures into software that will ‘stitch the faces together’. The software automatically removes my hairdo from each of the pictures. What is left are three pictures of my hairless face, sporting a serious expression. The PhD student then clicks a button in the software and we wait while it constructs the three 2D photos of my face into one coherent 3D facial depiction (Figure 2). It takes a few minutes. Then the virtual model of my face has been completed, and the PhD student demonstrates it is possible to rotate it and look at it from all angles. The ‘texture’ of my face can furthermore be removed, meaning all color is removed and a grey 3D mold remains. The PhD student promises to send us a 3D model of our faces in PDF format.

Our second stop in the lab is with facial shape, the genetically most complex of the traits discussed in this paper. In working towards the prediction of individualised faces, understanding the ‘genetic architecture’ of facial shape is deemed crucial (Claes and Shriver, 2016: 1; Roosenboom et al., 2016: 1), as ‘differences in relative size, shape and spatial arrangement (vertical, horizontal and depth) between the various facial features (e.g., eyes, nose, lips, etc.) make each individual human face unique’ (Richmond et al., 2018: 2). The particular lab we visited had already developed a kit to predict facial morphology. This kit, primarily based on the ‘summary statistics’ of ancestry and sex (Claes et al., 2014: 6), has been adopted by the US based Parabon Nanolabs, a company that specialises in the generation of ‘DNA-based’ facial composites. Being the only company to produce complete facial composites from DNA for criminal investigations, Parabon is unique in this sense. In the Netherlands, kits exist solely for the prediction of pigmentary traits and biogeographical ancestry.

To establish associations between genetic markers and facial morphology, large numbers of 3D faces are compared with DNA data. These 3D faces are gathered in datasets that are accessible online, containing up to thousands of 3D images. To test the effects of genes on these digital faces, they need to be made comparable. A network (referred to as a ‘mesh’) of dots is superimposed upon the faces. Each of these dots, known as ‘landmarks’, is connected to each other. As such, distances and curvatures on the facial surface can be captured. Each face is fitted with the mesh in such a way that they are identically landmarked, meaning ‘any given surface point on a face could be matched with the corresponding point on any other face’ (Crouch et al., 2018: E677). As explained in a study on facial shape by Crouch et al. (2018: E677), this is not unlike the symmetrising method that statistician and eugenicist Francis Galton developed in the late 1800s:

By using an approach that harks back to Galton’s (14) method for obtaining average faces, 14 landmark points, such as the tip of the nose, were marked on each facial image, which, through a process of mesh registration, enabled the overlaying of all images in relation to each other.

Through this process of overlaying and subsequent analysis, the average effects of genes on particular facial morphologies can be mapped. The landmarked faces, however, are not compared to one another at random. Researchers compare the faces of individuals whose facial traits are similar, and from there look for associations. One has to start somewhere. These physical similarities are assumed to exist within populations and, as such, individuals are assigned to a genetic population before comparison. In the case of the study conducted by Claes et al. (2014) the participants’ genetic ancestry was divided over two groups: West African and European. Genomic ancestry was then located in the shapes of the face:

For example, the area ratio shows increased surface area for the medial canthus, sides of the nose, and front of the chin on the European end of RIP-A [genomic ancestry] and a greater surface area for the nostrils and lips on the West African end of RIP-A. (Claes et al., 2014: 3)

RIP-A, a statistical variable that expresses genomic ancestry, became a means of ordering facial morphologies here. The researchers in this study sought to capture variation in facial shape through this variable, scalable from European to West African, making ancestry readable from the surface of the face: the eyes, the nose, the chin and the mouth. In addition, some individual faces in the study are then considered ‘typical’ of their genomic ancestry:

Despite the complex ways in which faces are affected by RIP-A and RIP-S, these variables are useful summaries of the degree to which particular faces are more or less ancestry-typical and sextypical, respectively. (Claes et al., 2014: 3)

Here the work of Galton, well-known for his method of extracting the ‘typical characteristics’ from a set of faces, surfaces again. Galton’s (1879) interest went out to facial similarities, to bring out ‘the average features of any given group’ (p. 133), which he accomplished by superimposing drawings of faces and combining them into aggregates. This method is not unlike the landmarking of faces using datapoints that I described above. Both practices rely on the alignment and superimposition of faces, and seek to produce representations ‘free of irregularities’ (Galton, 1879: 135). Similarly, in the above study we saw how researchers sought to bring out the average features of a European and West-African population. The genetic diversity that these groups encompassed were backgrounded, and their similarities given prominence. By assuming the existence of continentally defined (genetic) groups and translating these into averaged representations, race resurfaces as *type*. Any traces of individuality are erased as to make way for morphological similarities. During an interview, the researcher heading this laboratory explained the importance of removing these idiosyncrasies, or ‘errors’, before analysis.

XX: I mean, the point for us is to remove some error. Imaging artefacts, like, that guy has got, you know, an interesting beard. Just a little bit unusual. Most of the time we have no beards. We’re very careful about that. But this guy got in there somehow. But you’re basically shaving him because his beard is unusual. So there, I mean just to have a beard is unusual. But you can see it’s basically the same person.

ZZ: Mm-mm.

XX: In the projected face here the bangs were hanging down in their faces. And you know. Or, effectively by both trimming, removing some of that part, but, you know, systematically all the faces are trimmed the same way… So using a subset of all the variation removes some of the individual specific features of the face shape that were sometimes artefacts. Sometimes they could be, you know, a special kind of nose that *is* their nose, it’s not an imaging problem. But it’s something that is so unusual you are not seeing it in other people.

YY: You don’t want to see that in the projected face?

XX: It doesn’t add to your power to find genes. Because it’s something that is maybe unique to them.

The conversation demonstrates that individual particularities of faces get in the way of establishing associations between genetic loci and facial morphologies. Uniqueness is not productive in this research, as it is not generalizable to a broader population. Therefore the research is rather centred on distinguishing pheno- and geno*typic* groups. In moving away from individual traits and towards generalised (ancestry-determined) features we encounter the averaging work of Galton, but also the work of nineteenth century statistician Adolphe Quetelet. In Quetelet’s statistics the average figured prominently, as his ideal was to statistically capture the ‘average man’, mathematised from individuals to a mean: ‘the greater the number of individuals observed, the more do individual particularities, whether physical or moral, become effaced, and leave in prominent of view the general facts’ (Quetelet, 1842: 6).

The statistical approaches of Galton and Quetelet are mobilised quite explicitly in publications such as the one quoted above, but are folded into the analytical process of establishing associations between facial shapes and genetic markers in more intricate ways. Like Galton’s composite portraiture, these analyses are at the ‘intersection of photography and statistics’ (Sekula, 1986: 18). The ‘optical superimposition’ (Galton, 1879: 135) of 3D faces, with the ancestry averages this generates, brings about a result not unlike the racial types produced by Galton (Hopman and M’charek, 2020). To be sure, the statistics that inform contemporary research on facial morphology are based upon calculations and comparisons conducted with a speed and on a scale that would have been impossible in the times of Galton and Quetelet: ‘minute details of the face can be identified and compared across thousands of images in a few hours’ (White et al., 2019: 4).

Despite this drastic increase in computation power, by producing aggregate faces labelled by continental ancestry and stripped of idiosyncrasies these histories of composite portraiture and social statistics are brought into proximity. Taking into account the ultimate aim of FDP, coming as close as possible to the characterisation of individual suspects, the folding in of these particular histories is significant. Galton deliberately aimed at producing portraits of *types*, seeking to erase the particularities of individuals. Likewise, Quetelet interest was with the average: to statistically establish social regularities. Both scientists importantly wanted to move *away* from the individual, whereas contemporary FDP researchers exactly want to move *towards* it. Yet by visually and statistically averaging individual traits into group aggregates, Quetelet and Galton’s typological approaches find application in contemporary techniques, taking away from the promise of individuality this technology encompasses.

## Collection: The iris

**Figure 3.**
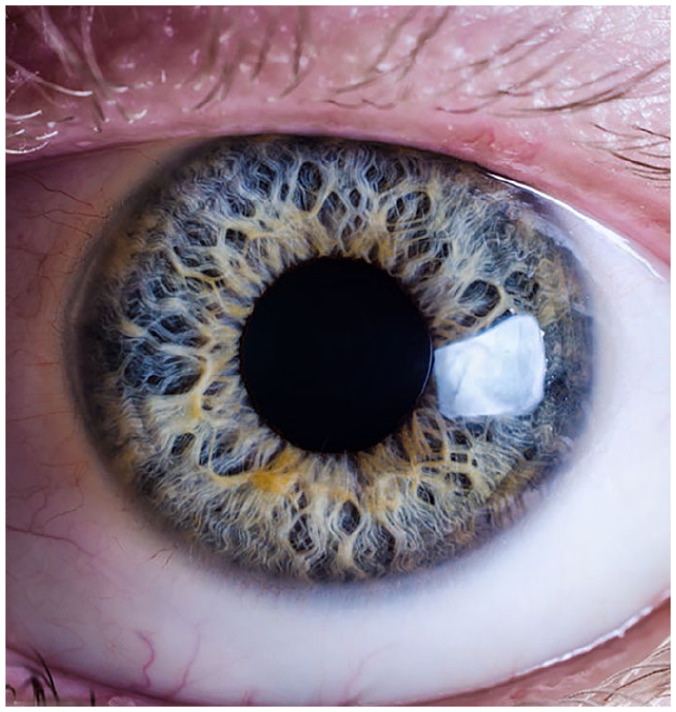
Close-up of a human iris. Source: Copyright Mathias Appel, Creative Commons, license https://creativecommons.org/licenses/by-sa/3.0/deed.en.

Participants are asked to put their chin in a holder right in front of the camera and are instructed to open their eyes as wide as possible. The PhD student explains that some participants cannot do this and have to hold their eyes open with their fingers. He then uses the high resolution camera to take a picture of their irises. The pictures are saved in a file on one of the computers. We walk over towards his computer and he opens the file to show me the images. They are surprisingly beautiful: seeing the eye up close reveals a vast variety. The variety is not limited to the color of the iris. Enlarged, the iris looks like a landscape, with what appear to be tiny rivers flowing through them (see Figure 3). Some have threadlike patterns, dots or even ‘holes’. The patterns seem to be different for each eye that we inspect. The PhD student continues to flip through the images, occasionally pointing out the individual particularities of the participants’ eyes. Some have small dark dots in their irises, or in just one of them, some have a yellow ring around their pupil. He goes through both the right and left eye of participants, demonstrating that up close, even per individual the eyes are not necessarily identical. I ask him what they will do with these pictures, as I wonder how they would be of use in a forensic investigation. The PhD student indicates he does not know yet how they will use this data, just that they are thinking about developing a method to map iris patterns. For now, they have collected images of the left- and right eye of 250 volunteers. A method for comprehensively mapping the wide variety of patterns visible in the irises does not yet exist. Developing such a model would be very complex, the PhD student explains, because establishing patterns is almost impossible. Until this becomes a possibility, the images therefore remain stored on the computer.

I want to finish this laboratory tour by going deeper into the promise of individuality. The previous two cases have primarily pointed at the pasts resonating in FDP research, yet studies of the iris importantly fold in themselves particular *futures*. Forensic genetic tests for the prediction of eye color were the first ones of their kind, as genetically, the color of the eyes is one of the simpler physical traits to predict (Kayser, 2015: 34). This is true especially for blue and brown eyes, which can be categorically predicted based on six genes, for example with the IrisPlex system, a tool developed by the Erasmus MC and Walsh laboratory in 2010|2011 (Kayser, 2015: 35). This system is the main phenotyping tool applied in Dutch investigations by the Netherlands Forensic Institute. The successes of predicting blue and brown eye color showed that translating genotypic into phenotypic information was possible. As a study on eye color reported: ‘Here, we used human eye (iris) color of Europeans as an empirical example to demonstrate that highly accurate genetic prediction of complex human phenotypes is feasible’ (Liu et al., 2009: R192). Studies on eye color were therefore seen as the ‘first steps forward in the creation of a fully individualised EVC [externally visible characteristics] prediction system for future use in forensic DNA intelligence’ (Walsh et al., 2011: 170). The case of the iris demonstrates the future-orientedness of FDP, promising a time to come in which individual faces can be predicted more fully and accurately.

In order to attain this future, the further accumulation of data on human phenotypic diversity is deemed essential (see Hopman, 2020): ‘We hope that the human pigmentation genetic community will continue to be productive and innovative *in their mission to harvest all DNA variation* underlying eye, hair, and skin colorations in modern humans’ (Liu et al., 2013: 572, emphasis added). FDP, I argue, is therefore importantly a *collecting* science. It relies on an accumulation of data on genetic and phenotypic variation. The iris, as such, can be taken as representative of FDP in general, where data is accumulated to provide predictions that are ever closer to the individual. During my fieldwork it became clear to me that this collection effort came with an earnest interest in human biological variation. The head of this US lab for example, a biological anthropologist, emphasised at a workshop in which I participated that he ‘had always been fascinated by human diversity’. He highlighted the collection efforts going on at his lab, stressing that they had already assembled 12,000 3D faces. A colleague asked him what other kinds of data they were gathering. The biological anthropologist replied: ‘We have 3D scans, do photogrammetry, texture maps, collect skin color measurements, hair samples, actual hair cuttings for microscopic analysis, voice recordings, and strength measures’. His colleague, amazed, complimented him on his data: ‘To have data is beautiful. You can always hypothesise and run regression analyses. This is beautiful data’.

The data-centredness of these practices resonates with Kohler’s (2007) description of nineteenth century collection sciences. He writes that for biological taxonomy, for example, the collection of data in the shape of physical objects was the only means to further research on the variation of species, making collection practices crucial to progress (p. 437). Likewise, for collecting sciences such as archaeology and palaeontology, Kohler (2007: 446) observes that as part of a ‘trend towards empirical exactness’, ‘more abundant and precise data’ were needed. A similar drive is visible in FDP research. Data is collected with the goal of increasing precision through abundance. Whereas eye *color* variation in itself is substantial, researchers are turning their attention from categorical predictions of color to include crypts, furrows, pigmentation spots and nodules. With the collection of these data comes the promise that, ‘over time, predictions will get more precise’ (Lippert et al., 2017: 10169).

Classical physical anthropology was also importantly a collection science. Sysling (2016) demonstrates the salience of collection to this field in her work on Dutch physical anthropologists conducting research in the West Indies during the nineteenth and twentieth centuries. During this period, researchers assembled large collections of data on human difference. In one of her chapters Sysling quotes the anthropologist Kleiweg de Zwaan (1915), who emphasised in a journal piece that ‘As a rule it is advised to collect as much material as possible, because the more objects that are available for study, the more valuable are the results of that research’ (p. 412). Not unlike FDP researchers encouraging their colleagues to harvest DNA variation, these Dutch physical anthropologists in Indonesia stimulated each other to accumulate data: They collected measurements, skulls and bones, made drawings and scribbled in notebooks. As Kohler (2007: 447) pointedly summarises: ‘All that stuff!’ The gathered specimens and measurements were to provide these anthropologists with the scientific evidence to support theories about the existence of differentiable human races (Sysling, 2016: 9; also El-Haj, 2007: 286), an undertaking that was not straightforward. Rather than presenting the researchers with human varieties that could be clearly delineated into racial types, they were overwhelmed by the diversity of the collected objects and measurements. Many anthropologists therefore left it to future researchers to sort out the implications of their data (Sysling, 2016: 127). Importantly, these data were thus promissory with regard to their ‘eventual usefulness’ (Sysling, 2016: 171) towards distinguishing racial types.

Likewise, developing prediction models based on the contemporary iris data was difficult. The highly variable irises were, borrowing words from Daston and Galison (1992) describing the standardisation of natural objects in the late nineteenth century sciences (p. 85), ‘too plentiful and too various … too quirkily particular to cooperate in generalizations and comparisons’ to create a comprehensive prediction model. Yet like anthropological data, the iris photographs held the promise that, in time, with the expansion of knowledge and the development of new technologies, researchers will be able to develop the right prediction models. Until then, the irises remain saved in a computer file. Radin’s (2013) work on the ‘latent potential’ of blood samples is helpful here. Biologists in the 1960s and 1970s were unable to analyse these samples with then-existing methods. The biologists, however, foresaw a future in which these samples would become useful. Like the iris data, the samples possessed ‘the potential to yield new knowledge about biological variation as novel analytical techniques were developed’ (Radin, 2013: 488). Iris images are expected to provide insights into human variation *in time*.

Temporality, in the case of the iris, thus trickles down as an imaginary of the future. In this it is exemplary of FDP technologies more generally, folding into itself a future in which individuality becomes achievable. The iris, in all its details, can be taken as a microcosm of the genetically unique individual (hence its suitability for identification purposes). As such, FDP is part of a broader trend in studies of human diversity, in which a ‘shift away from typological thought has come [with] an increased interest in individuals and what genetics can tell us about the unique identity and history of each person’ (Bolnick, 2008: 70). Being able to predict the genetics behind an individual’s face was in fact described as the ‘Holy Grail’ of appearance prediction at different moments during my fieldwork.

As we have already seen in the cases of skin pigmentation and facial morphology, individual idiosyncrasies are not productive in phenotyping research. In the end, the research demands populations and categorisations of them. Individuality, the ‘Holy Grail’ of this field, slips away in the increasing complexity of the accumulated data. While geneticists working on FDP have no stakes in establishing racial patterns of phenotypic variation, it is precisely in the generation of increasing amounts of data and the categorisations they require that we encounter race. Here the field of FDP bears resemblance to earlier physical anthropology, as both fields function by a logic of ‘exuberance’ (Haraway, 1997: 234). Addressing twentieth century racial taxonomy, Haraway (1997: 234) stresses:

In these taxonomies, which are, after all, little machines for clarifying and separating categories, the entity that always eluded the classifier was simple: race itself. The pure Type, which animated dreams, sciences, and terrors, kept slipping through, and endlessly multiplying, all the typological taxonomies.

Likewise, we have seen how in the field of FDP individuality always ‘eluded’ classification. Both the search for biologically distinguishable racial types and for characterisations of the individually unique face, although practically unattainable, were held out as central goals of these fields, and fed into data-collection and analysis practices to which racial classifications became essential.

## Concluding discussion

In the development of FDP technologies, a progressive, linear temporality and the steady move away from race cannot adequately capture the temporal complexities of this research. Even though these technologies may be progressing in terms of level of detail, storage capacity and computing time, physical anthropological and statistical histories are simultaneously folded into FDP practices in intricate ways, entangling race in novel configurations. By accounting for these histories (and futures!) it becomes possible to attend to the politics of these practices as they are done ‘in the here and now’ (M’charek, 2014: 48), and to allow for a consideration of race as ‘an effect of temporal cuts and folds’ (M’charek and van Oorschot, 2019: 242). This means that although the technology is presented as neutral and progressive, having moved beyond race (see Benjamin, 2019), in the development of FDP kits, techniques entangled with race, racism and their histories can be unfolded.

The folds presented in this article are in part the consequence of the choices of the author, who traced the histories of technologies through references in research papers, read about physical anthropological methods and asked questions in laboratories. Yet in some instances the practitioners conducting research on FDP explicitly mobilised these pasts as well: in, for example, the reproduction of the von Luschan skin measurement technique, the foregrounding of Biasutti’s global skin color distribution map in a contemporary study on skin pigmentation, and the reliance on Galton’s method of facial landmarking in a contemporary study on facial shape.

Taking the historicity of these techniques seriously while at the same time remaining attentive to the specificities of contemporary technologies and practices, requires temporally sensitive methods. I have proposed an analysis based on the notion of the fold to achieve this, and have traced the historical resonances in present practices to deal with these complexities, adding a temporal understanding to the existing body of literature on FDP and race (Cole and Lynch, 2006; Ossorio, 2006; Skinner, 2020; Wienroth et al., 2014). In the skin case, variation was quantified through the measurement of light-reflectance, producing a specificity that needed to be ordered through visual comparison. Here, race materialised in the transformation of quantitative measurements into workable categories, building on ‘eighteenth-century perceptual regimes’ (Gilroy, 1998: 844) able to read race directly from the surface of the skin. In the second case, race became measured out through the generation of population averages and the distillation of ancestry-typical facial features from digitally landmarked faces. Finally, data on the iris brings into view a fascination with human diversity, folding in physical anthropological collection practices.

Several authors have argued that science is moving away from race as a quality of the body. Race, they argue, is still relevant, but it has become molecularised (Duster, 2006; El-Haj, 2007; Fullwiley, 2007). Rothman (1998: 64–65) sums this up:

No longer visible, no longer divisible, race has moved inward from body to blood to genes; from solid to liquid to a new crystallization. Race is now a code to be read; the science of race is the science of decoding. The discriminating, discerning, trained eye that can recognize the ‘essential’ or defining characteristics in the individual that confer race categorization will now be looking not at the face, the angle of the cheek, the color of the skin, but at the DNA.

Yet my praxiographic analysis of the three cases demonstrates that in FDP, race is enacted as a matter of both the bodily surface and of the genome. In phenotyping practices, the face, the angle of the cheek and the color of the skin have become markers of race par excellence. With M’charek (2020: 372) I argue that the phenotype has made a return in forensics, making race into ‘a matter of sur-face’. It is not only that the surface of the body in general is gaining traction, strikingly it is the *same* physical characteristics that scientists are focusing their attention towards. As physical anthropologist Rowe (1950: 211) wrote on how to determine a race by looking at physical characteristics: ‘The physical anthropologist would like to know the color of the skin, eyes, and hair, the shape of the head, face, and nose, stature and many other things about the individual’. Strikingly, 70 years later these are the exact same traits geneticists are seeking to predict from DNA. As observed by Sysling (2016: 180): ‘many of the techniques and ideas behind physical anthropology continued under other guises, such as human biology [anthropobiologie], forensic anthropology and DNA research’.

The case of FDP is uniquely suited to show how scientific pasts are implicated in contemporary, emerging technologies, asking for temporally and praxiographically sensitive methods that can attend to them and their consequences. Importantly, this analysis points out that even though FDP technologies are presented as neutral and as having moved beyond race, particular histories, techniques and objects entangled with race and racism are folded into the research practices surrounding the prediction of physical appearance from DNA. Whereas researchers may be aware of these histories, and while they may be articulated in some studies, when facial renderings that result from FDP analyses travel from laboratories into criminal investigations and further into society these histories (and their continuing relevance) become obscured, ‘pushed into invisibility out-there, in the final product when suddenly all the intermediate steps are made to disappear’ (Law, 2004: 84). FDP therefore demonstrates the necessity of historical knowledge when considering current practices, and the futures that we envision.
